# Effects of a Four-Week Combined Respiratory Muscle Training and Breathing Exercise Program on Breath-Holding Time, Functional Performance, Sleep Quality, and Perceived Stress in Healthy University Students

**DOI:** 10.3390/jcm15135151

**Published:** 2026-07-02

**Authors:** Asma Alonazi, Lena Almasoudi, Najd Almzini, Najla Alzakari, Renad Almutairi, Saud Alhassan, Ahmed Albosager, Qasem Alsaeed, Ali Alojayan, Abdullah Alismail

**Affiliations:** 1 Department of Physical Therapy and Health Rehabilitation, College of Applied Medical Sciences, Majmaah University, Riyadh 11952, Saudi Arabia; lenaalmasoudi@outlook.sa (L.A.); najdalmzini@outlook.com (N.A.); najla.alzakari8@gmail.com (N.A.); renad.almutaiiri@gmail.com (R.A.); saud.rf.alh@gmail.com (S.A.); saqerya2003@gmail.com (A.A.); qasemabdullah1423@gmail.com (Q.A.); aliojayan23@gmail.com (A.A.); 2 Research and Innovation Institute, Ministry of Defense Health Services, Riyadh 12426, Saudi Arabia; dr.abdullah.alismail@gmail.com; 3 Department of Cardiopulmonary Sciences, Rush University, Chicago, IL 60612, USA; 4 Department of Medicine, School of Medicine, Loma Linda University, Loma Linda, CA 92354, USA

**Keywords:** breathing exercises, respiratory muscle training, functional performance, sleep quality, stress, university students

## Abstract

**Background:** Breathing exercises and respiratory muscle training (RMT) are accessible, low-cost interventions that may improve both physical and psychological health through respiratory and autonomic regulation. Despite growing interest in respiratory interventions, their combined effects on physical performance, sleep quality, and perceived stress in healthy university students remain poorly understood. **Methods:** This pilot pre-post experimental study was conducted in Saudi Arabia. Students aged 18–25 years with a BMI < 29.9 kg/m^2^ participated in a 4-week intervention consisting of diaphragmatic breathing, pursed-lip breathing, and threshold-loaded inspiratory and expiratory muscle training (40–60% maximal inspiratory pressure), performed five days per week. Outcomes assessed before and after the intervention included chest expansion, breath-holding time, Timed Up-and-Go (TUG), Sit-to-Stand (STS), Pittsburgh Sleep Quality Index (PSQI), and Perceived Stress Scale (PSS). Paired statistical analyses and correlation tests were performed. **Results:** Forty participants (50% female; median age 22 years; BMI 23.14 ± 4.05 kg/m^2^) completed the study. Breath-holding time increased by 132% (*p* < 0.001, r = 0.87). Functional performance improved significantly, with TUG decreasing by 9% (d_z = 1.01) and STS improving by 12% (d_z = 1.12) (*p* < 0.001). Sleep quality improved by 26%, while perceived stress decreased by 19% (*p* ≤ 0.001). Significant exploratory correlations were observed between TUG and STS change scores (r = 0.49), PSQI and PSS changes (r = 0.33), and STS and PSS changes (r = 0.33). **Conclusions:** Participation in a four-week combined breathing exercise and respiratory muscle training program was associated with favorable within-group changes in breath-holding time, functional performance, sleep quality, and perceived stress among healthy university students. Given the single-arm design, these findings should be considered preliminary and hypothesis-generating. Randomized controlled studies are needed to determine efficacy and causal relationships.

## 1. Introduction

The transition to university life represents a critical developmental window during which health behaviors are established, with profound implications for long-term well-being [1]. University students face escalating social demands, academic pressures, and competitive environments that place them at high risk of compromised physical and psychological health [2]. This population is particularly vulnerable to sleep deprivation, stress, anxiety, and depressive symptoms, all of which may adversely affect academic engagement, productivity, and long-term professional development [3,4].

Concurrently, lifestyle changes associated with economic development and increased sedentary behavior have contributed to declining physical fitness levels among university students globally [5,6]. Suboptimal physical fitness from young adulthood through middle age significantly increases the risk of chronic, non-communicable diseases later in life, including cardiovascular disease, type 2 diabetes, cancer, and stroke [7,8]. A systematic review by Ruiz et al. [9] demonstrated a strong association between cardiorespiratory fitness during childhood and adolescence and favorable cardiovascular health outcomes in adulthood, underscoring the critical importance of early health promotion strategies during the university years.

University students are increasingly exposed to sedentary lifestyles characterized by prolonged sitting, screen use, and reduced participation in physical activity. Recent evidence indicates that insufficient physical activity and sedentary behavior are highly prevalent among university students globally and are associated with poorer physical and mental health outcomes [3,4,5,6]. These lifestyle behaviors are also increasingly reported among young adults in Saudi Arabia, where rapid socioeconomic development and technological advancement have contributed to reduced daily physical activity levels. Such trends highlight the need for accessible, low-burden interventions that can be integrated into students’ daily routines and promote both physical and psychological well-being.

While physical activity promotion remains the cornerstone of preventive health recommendations, adherence to structured exercise programs among university students is notoriously low due to time constraints, lack of motivation, and competing academic demands [10,11]. This implementation gap necessitates the development of low-cost, low-burden, easily adoptable interventions that can be integrated into daily routines without requiring substantial time commitments or specialized facilities.

An underexploited avenue for intervention targets the fundamental role of the respiratory system in integrating physiological and psychological functioning. The respiratory muscles, particularly the diaphragm, serve dual functions: they are essential for ventilation and also play a critical role in postural control and trunk stability [12]. Under conditions of respiratory load or compromise, diaphragmatic function may prioritize ventilation over postural support, potentially compromising balance and functional performance [13,14].

Furthermore, respiratory patterns directly modulate autonomic nervous system activity. Slow, controlled breathing has been associated with enhanced parasympathetic activity and reduced sympathetic activation, thereby mitigating physiological stress responses [13,15]. This autonomic regulation extends to sleep quality, as sleep architecture is closely linked to neuroendocrine activity, particularly circulating catecholamine levels [13,16]. Respiratory muscle training (RMT) may therefore improve sleep quality by reducing sympathetic activation and lowering circulating catecholamine levels, a mechanism objectively demonstrated in patients with obstructive sleep apnea [17].

Breathing exercises and RMT thus represent directed interventions that address the interconnected domains of respiratory efficiency, postural control, autonomic regulation, and stress modulation. These interventions have demonstrated efficacy in improving respiratory muscle strength, physical endurance, and stress regulation across various clinical and athletic populations [14,15,18]. However, the potential benefits of combined breathing exercise and RMT programs on both physiological and psychological outcomes have been insufficiently explored in healthy university-aged populations, particularly within the Saudi context where sedentary lifestyles are increasingly prevalent.

Importantly, the rationale for respiratory muscle training in the present study differs from its traditional use in pulmonary rehabilitation. Although healthy university students generally do not exhibit respiratory muscle weakness, respiratory muscle conditioning has increasingly been investigated as a means of optimizing physiological performance, exercise capacity, and autonomic regulation in healthy individuals and athletes. The present pilot study therefore aimed to explore whether similar adaptations could extend beyond respiratory outcomes to functional performance, sleep quality, and perceived stress in a healthy university population.

Breathing exercises and RMT training target different but complementary physiological mechanisms. Breathing exercises such as diaphragmatic breathing and pursed-lip breathing primarily aim to improve breathing patterns, reduce respiratory rate, enhance parasympathetic nervous system activity, and promote relaxation. In contrast, respiratory muscle training uses threshold loading to increase inspiratory and expiratory muscle strength and endurance, thereby improving respiratory efficiency and reducing respiratory muscle fatigue.

The respiratory muscles, particularly the diaphragm, serve dual functions: they are essential for ventilation and also play a critical role in postural control and trunk stability [12]. Under conditions of respiratory load, diaphragmatic resources may be preferentially allocated toward ventilation rather than postural support, potentially influencing movement efficiency and functional performance [13,14]. Although healthy university students do not typically exhibit clinically significant balance impairments, optimization of respiratory muscle function may contribute to improved movement efficiency and functional task performance.

We propose that a multimodal intervention combining breathing exercises and RMT can improve respiratory muscle efficiency, which may: (1) reduce the metabolic cost of breathing during daily activities, (2) free up respiratory muscle resources for postural control (enhancing functional performance), and (3) directly modulate autonomic balance to improve sleep quality and reduce perceived stress.

Therefore, the primary objective of this pilot study was to evaluate within-group changes of a four-week breathing exercise and respiratory muscle training program on breath-holding time, a composite respiratory performance measure influenced by respiratory function, ventilatory control, and behavioral factors, functional performance (Timed Up-and-Go and Sit-to-Stand tests), sleep quality (PSQI), and perceived stress (PSS), among university students in Saudi Arabia. Secondary objectives were to explore associations among outcome changes and examine potential gender differences in response to the intervention.

## 2. Materials and Methods

### 2.1. Ethics Approval

Ethical approval was obtained from the Institutional Ethics Committee of Majmaah University, Al Majma’ah, Riyadh, Saudi Arabia (MUREC-Dec.15/COM-2024/99) prior to initiating the research. All participants provided written informed consent after receiving a thorough explanation of the study objectives and procedures. Participant anonymity and confidentiality were strictly maintained throughout the study.

### 2.2. Study Design and Setting

This study was designed as a single-arm pilot pre-post intervention study to assess the impact of a four-week breathing exercise and RMT program on breath-holding time, physical performance, sleep quality, and perceived stress levels among university students. This single-group pilot design was selected to evaluate feasibility, establish preliminary effect sizes, and examine inter-relationships among outcomes prior to conducting larger controlled trials. The intervention lasted four weeks, with assessments conducted one week before and within three days following program completion. The study was conducted at Majmaah University in a controlled, quiet, well-ventilated indoor setting to ensure standardization during assessments. All tests and exercises were performed under supervision during designated hours.

### 2.3. Study Participants

Participants were recruited through university announcements and health awareness events using a convenience sampling approach. Eligibility criteria included: (1) age 18–25 years, (2) current enrolment as a university student, and (3) BMI < 29.9 kg/m^2^. Exclusion criteria included: (1) diagnosed respiratory conditions (asthma, COPD, etc.), (2) diagnosed cardiovascular conditions, (3) diagnosed mental health disorders requiring treatment, (4) recent surgery (within 6 months), (5) current smoking, and (6) regular participation in structured exercise or breathing programs. An initial screening using a standardized health questionnaire was conducted to confirm eligibility.

### 2.4. Sample Size

Sample size was determined a priori using a two-tailed paired samples *t*-test (dz = 0.50; α = 0.05; 1 − β = 0.80). This indicated a minimum of 34 participants needed for the study; N = 40 were enrolled. A sensitivity analysis indicated that this sample provided 80% power to detect a within-subject effect of dz ≥ 0.45.

### 2.5. Assessment Tools and Procedures

All assessments were conducted by the same trained assessor who was not blinded to the time point (pre vs. post), representing a limitation acknowledged in Section 4.2.

#### 2.5.1. Anthropometric Measurements

Height was measured using a standard stadiometer (Seca, Hamburg, Germany) to the nearest 0.1 cm. Weight, and muscle mass were measured using a calibrated digital bioelectrical impedance scale (Tanita BC-418, Tokyo, Japan). BMI was calculated using the standard formula (kg/m^2^).

#### 2.5.2. Chest Circumference

Chest circumference was measured using a flexible, non-stretchable measuring tape at the level of the fourth intercostal space during maximal inspiration and maximal expiration, following standardized procedures [19]. Measurements were recorded in centimeters, and chest expansion was calculated as the difference between inspiratory and expiratory circumferences. Three measurements were taken, and the average was used for analysis.

#### 2.5.3. Breath-Holding Time

Breath-holding time (voluntary apnea time) was defined as the duration from maximal inhalation to the point at which the urge to breathe exceeded the participant’s ability to continue holding the breath [20], which represents a composite measure influenced by respiratory muscle function, ventilatory control, and psychological tolerance. Participants were seated comfortably and instructed to perform a maximal inhalation and then hold their breath for as long as possible. Time was recorded in seconds using a digital stopwatch. A single trial was performed to avoid the influence of learning effects or fatigue.

#### 2.5.4. Timed up and Go Test (TUG)

The TUG test is a validated clinical assessment of functional mobility and dynamic balance [21]. Participants were instructed to rise from a seated position (standard armless chair, seat height 46 cm), walk forward 3 m, turn around, return to the chair, and sit down. The time from the command “go” to returning to the seated position was recorded in seconds using a digital stopwatch. One practice trial was followed by two test trials, with the average time used for analysis.

#### 2.5.5. Sit-to-Stand Test (STS)

The five-repetition STS test was used to assess lower-limb functional strength and postural control [22]. Participants performed five consecutive sit-to-stand repetitions as quickly as possible from a standard-height chair (seat height 46 cm), with arms crossed over the chest. The time from the initial command to the final standing position after the fifth repetition was recorded. One practice trial was followed by two test trials, with the average time used for analysis.

Although commonly used in clinical and geriatric populations, the TUG and STS tests have also been applied in healthy young adults as practical measures of functional mobility, movement efficiency, and lower-limb functional performance. The purpose of including these tests was not to identify mobility impairments but rather to evaluate whether respiratory training could influence functional task performance in a healthy population.

#### 2.5.6. Pittsburgh Sleep Quality Index (PSQI)

The PSQI is a self-administered questionnaire evaluating sleep quality and disturbances over the preceding month [23]. It comprises 19 items generating seven component scores (subjective sleep quality, sleep latency, sleep duration, habitual sleep efficiency, sleep disturbances, use of sleep medication, and daytime dysfunction), which sum to a global score ranging from 0 to 21. Higher scores indicate poorer sleep quality, with scores > 5 indicating clinically significant sleep disturbance. The Arabic version of the PSQI has been validated for use in Saudi populations [24].

#### 2.5.7. Perceived Stress Scale (PSS)

The 10-item PSS was used to assess perceived stress over the preceding month [25]. Participants responded to items on a 5-point Likert scale (0 = never to 4 = very often), with total scores ranging from 0 to 40. Higher scores indicate greater perceived stress. The Arabic version of the PSS has demonstrated good reliability and validity [26].

### 2.6. Intervention Program

Participants completed a structured 4-week breathing exercise and RMT program performed five days per week (Monday through Friday). All sessions were supervised by trained research assistants to ensure correct technique and adherence. Participants maintained daily training logs documenting exercise completion, symptoms, and any difficulties encountered. Participants demonstrating less than 80% adherence (fewer than 16 of 20 sessions) were excluded from the final analysis.

Initial IMT and EMT loads were determined individually using baseline MIP and MEP measurements. Training loads were maintained within the target range of 40–60% of baseline MIP or MEP throughout the four-week intervention. MIP and MEP were not reassessed during the intervention; therefore, progressive load adjustment was not performed.

Adherence was monitored using supervised attendance records and participant training logs. Participants documented session completion, symptoms, and any training-related difficulties. No adverse events or clinically significant discomfort requiring discontinuation were reported.

#### 2.6.1. Breathing Exercises (20 min/Day)

Breathing exercises were performed twice daily (morning and evening), each session lasting 10 min.

Diaphragmatic breathing (5 min per session): Participants were instructed to assume a supine position with knees flexed and one hand placed on the abdomen. They were instructed to inhale slowly through the nose, allowing the abdomen to rise, followed by controlled exhalation through pursed lips, allowing the abdomen to fall. The breathing ratio was maintained at approximately 1:2 (inhalation: exhalation).

Pursed-Lip Breathing (5 min per session): Participants performed nasal inhalation followed by prolonged exhalation through pursed lips, maintaining an inspiratory: expiratory ratio of 1:3. This technique was practiced in a seated position to reduce respiratory rate and prolong exhalation.

#### 2.6.2. Respiratory Muscle Training (10–15 min/Day)

RMT was performed once daily using threshold-loading devices.

#### 2.6.3. Inspiratory Muscle Training (IMT)

Participants used a threshold IMT device (POWERbreathe Medic, HaB International, Southam, UK) set at 40–60% of their individual maximum inspiratory pressure (MIP). MIP was measured at baseline using a handheld respiratory pressure meter (MicroRPM, CareFusion, Chatham Maritime, UK) following standardized guidelines [25]. Participants performed two sets of 10 resisted inhalations, with a 1 min rest between sets. 

#### 2.6.4. Expiratory Muscle Training (EMT)

Participants used a threshold EMT device (EMST 150, Aspire Products, LLC, Cape Carteret, NC, USA) set at 40–60% of their individual maximum expiratory pressure (MEP). Participants performed two sets of 10 resisted exhalations, with a 1 min rest between sets.

### 2.7. Statistical Analyses

Data were analyzed using IBM SPSS Statistics 31. Normality was assessed with the Shapiro–Wilk test (α = 0.05). Continuous data are presented as mean ± SD or median (IQR), and categorical data as frequencies (%). Pre-post changes were analyzed using paired *t*-tests (parametric) or Wilcoxon signed-rank tests (non-parametric), with effect sizes reported as Cohen’s d_z (for paired *t*-tests) and r = |Z|/√N (Rosenthal; for Wilcoxon signed-rank tests), with the parametric versus non-parametric choice based on the Shapiro–Wilk normality of each change score. Gender differences in change scores were examined using independent *t*-tests or Mann–Whitney U tests (with effect sizes expressed as Cohen’s d and rank-biserial correlation, respectively). Correlations between change scores were assessed using Pearson or Spearman coefficients. Associations between baseline variables and outcomes were also explored. All tests were two-tailed (α = 0.05). No correction for multiple comparisons was applied due to the exploratory nature of the study.

## 3. Results

### 3.1. Participant Flow and Baseline Characteristics

A total of 47 university students were screened for eligibility. Seven were excluded (BMI > 29.9 kg/m^2^ [*n* = 3], current smoking [*n* = 2], diagnosed asthma [*n* = 2]), and 40 participants enrolled and completed the full intervention. Adherence was excellent, with all 40 participants completing at least 18 of 20 sessions (90% adherence); therefore, no participants were excluded from the final analysis.

The sample comprised equal numbers of male and female participants (50% each). Median age was 22 (IQR: 22–23) years, with a range of 19-24 years. Baseline anthropometric and outcome measures are presented in Table 1. Notably, the mean baseline PSQI score (5.90 ± 2.99) approached the clinical cutoff for poor sleep quality (>5), indicating that this sample exhibited mildly impaired sleep at baseline, providing meaningful room for improvement.

### 3.2. Intervention Effects on Primary and Secondary Outcomes

Table 2 presents pre- and post-intervention comparisons for all outcomes. The primary outcome, breath-holding time, demonstrated a dramatic and statistically significant improvement, more than doubling from a median of 21.36 s at baseline to 49.48 s post-intervention (median increase: +27.12 s, *p* < 0.001, r = 0.87). This represents a 132% increase in breath-holding time.

All secondary outcomes also showed statistically significant improvements. Functional performance improved substantially: TUG completion time decreased by 9% (8.18 ± 1.12 to 7.42 ± 1.17 s, mean difference: −0.75 s [95% CI: −0.99, −0.51], *p* < 0.001, d_z = −1.01), and STS completion time decreased by 12% (10.12 ± 1.89 to 8.90 ± 1.50 s, mean difference: −1.21 s [95% CI: −1.56, −0.87], *p* < 0.001, d_z = −1.12). Both effect sizes are considered large.

Psychological outcomes also demonstrated statistically significant within-group changes. PSQI global scores improved by 26% (5.90 ± 2.99 to 4.35 ± 2.35, mean difference: −1.55 points [95% CI: −2.43, −0.67], *p* = 0.001, d_z = −0.56), moving the sample mean below the clinical cutoff for poor sleep quality. PSS scores improved by 19% (15.78 ± 6.02 to 12.80 ± 5.34, mean difference: −2.98 points [95% CI: −4.53, −1.42], *p* < 0.001, d_z = −0.61). Both effect sizes are medium-to-large.

Chest expansion showed a small, non-significant increase (median change: 0.00 cm, *p* = 0.140). As expected, anthropometric variables (weight, BMI, muscle mass) remained unchanged over the 4-week intervention period, confirming that observed improvements were not attributable to changes in body composition.

### 3.3. Relationships Among Intervention-Induced Changes

**Figure 1:**
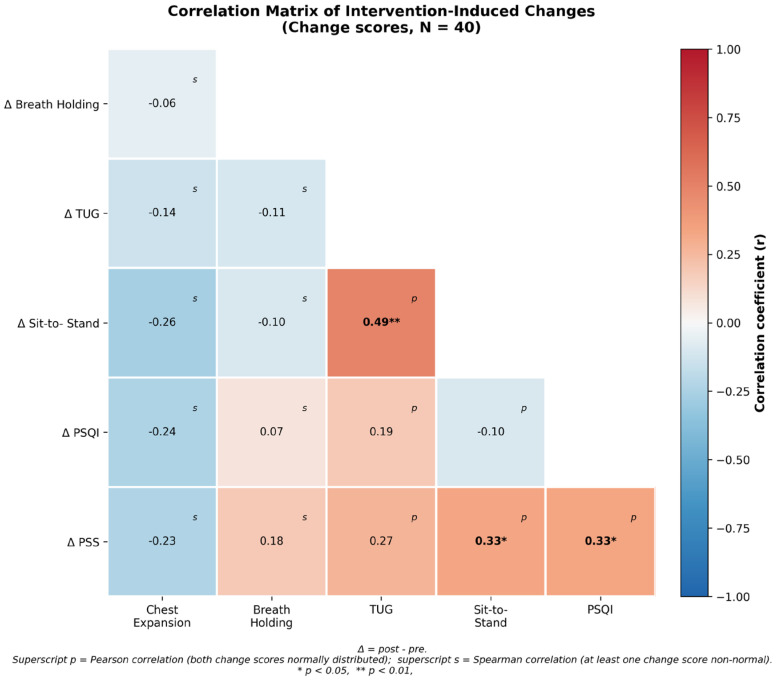
Exploratory correlation matrix of outcome change scores (Δ = post − pre; N = 40). Values represent Pearson’s r (superscript *p*) or Spearman’s ρ (superscript s) according to data distribution. Significant correlations are indicated by * *p* < 0.05 and ** *p* < 0.01.

Correlation analyses using change scores revealed three significant relationships that identify exploratory associations among change scores (Figure 1).

First, improvements in the two functional performance measures were strongly and positively correlated (TUG and STS: r = 0.49, *p* = 0.001). Participants who showed greater improvements in dynamic balance (TUG) also demonstrated greater improvements in lower-limb strength/power (STS), suggesting consistency between the two functional performance measures.

Second, improvements in sleep quality and perceived stress were moderately correlated (PSQI and ΔPSS: r = 0.33, *p* = 0.036). Better sleep quality was associated with greater stress reduction, suggesting these psychological benefits may share a common pathway.

Third, and most notably, improvements in STS performance were moderately correlated with stress reduction (STS and ΔPSS: r = 0.33, *p* = 0.041). This cross-domain correlation between physical function and psychological well-being suggests that changes in these domains may be related. However, the underlying mechanisms were not assessed in the present study.

Breath-holding time changes were not significantly correlated with changes in any other outcome (all *p* > 0.05), suggesting that breath-holding endurance improvements may represent a distinct domain of adaptation. Baseline characteristics (age, BMI, weight, muscle mass) did not predict intervention response for any outcome (all *p* > 0.05).

### 3.4. Gender Subgroup Analysis

Table 3 presents gender-specific change scores. Only one outcome demonstrated a significant gender difference: males experienced approximately 40% greater improvement in breath-holding time compared to females (+28.67 ± 8.99 vs. +20.47 ± 10.27 s, U = 284, *p* = 0.023, r = −0.42). This medium-to-large effect size suggests that males may derive greater breath-holding endurance benefits from the intervention. No significant gender differences were observed for chest expansion, TUG, STS, PSQI, or PSS changes (all *p* > 0.05).

## 4. Discussion

This pilot study examined the within-group changes associated with a four-week breathing exercise and respiratory muscle training program on breath-holding endurance, functional performance, sleep quality, and perceived stress among university students in Saudi Arabia. The findings demonstrate that this low-cost, low-burden intervention produced clinically meaningful improvements across multiple health domains: breath-holding time more than doubled (+132%), functional mobility improved by 9-12%, sleep quality improved by 26%, and perceived stress decreased by 19%. The significant correlations among improvements in functional performance and psychological well-being suggest that some intervention-related changes may be interconnected; however, the observed associations were moderate in magnitude and should be interpreted as exploratory rather than evidence of a unified mechanistic response.

Because participants did not present with respiratory disease or respiratory muscle weakness, the purpose of this intervention was not therapeutic rehabilitation but physiological conditioning. Similar to strength or endurance training applied in healthy individuals, respiratory muscle training may improve physiological reserve even in the absence of baseline impairment. Nevertheless, whether these adaptations translate into meaningful health benefits in healthy university students remains uncertain and requires confirmation in randomized controlled trials.

The observed associations among functional and psychological outcomes provide preliminary support for the hypothesis that some intervention-related benefits may share common physiological pathways. However, these mechanisms remain speculative and require confirmation using objective physiological measures in future studies. Breath-holding time increased by 132% following the intervention, indicating a marked improvement in this composite respiratory performance measure. Because breath-holding time is influenced by multiple physiological and behavioral factors, this finding should not be interpreted as direct evidence of respiratory muscle adaptation. Breath-holding reflects multiple factors, including lung volume, metabolic rate, chemoreflex sensitivity, respiratory muscle efficiency, and psychological tolerance to dyspnea [20].

The substantial increase observed in breath-holding time should be interpreted cautiously. Although breath-holding performance may be influenced by respiratory muscle function, it is also affected by multiple physiological and behavioral factors, including motivation, tolerance to respiratory discomfort, chemoreflex sensitivity, anxiety, lung volume at test initiation, and familiarity with the testing procedure. Therefore, the observed increase should not be interpreted as direct evidence of respiratory muscle adaptation. Rather, breath-holding time should be considered a composite respiratory performance measure. Furthermore, because post-intervention MIP and MEP measurements were not obtained, objective changes in respiratory muscle strength or endurance could not be confirmed.

These improvements may reflect enhanced respiratory muscle performance, improved breathing efficiency, repeated exposure to breath-holding, or other physiological and behavioral adaptations; however, the present study did not directly assess these mechanisms. The observed association between improvements in functional performance and reductions in perceived stress suggests a potential relationship between physical and psychological responses to the intervention [14,15,27].

Consistent with previous studies, breathing interventions improve respiratory function, although the larger effect observed here may be attributed to threshold-loaded RMT [15,28]. The lack of change in chest expansion indicates that gains were driven by muscle function and efficiency rather than thoracic mobility.

Improvements in TUG (9%) and STS (12%) suggest enhanced functional performance and movement efficiency; however, these findings should be interpreted cautiously because both tests may exhibit ceiling effects in healthy young adults, assessor blinding was not feasible in the present study, and the clinical relevance of the observed changes remains uncertain because minimal clinically important difference thresholds have not been established for this population.

This may be explained by the diaphragm’s dual role in respiration and postural control [12]. One possible explanation is that improved diaphragmatic efficiency through RMT may reduce competition between breathing and trunk stabilization; however, this mechanism was not directly assessed [29]. Similar improvements have been reported following IMT in clinical populations, including better TUG performance and postural stability [30,31].

Because breathing exercises and respiratory muscle training were delivered simultaneously, the present study cannot determine the independent contribution of each intervention component. Future studies employing factorial or multi-arm randomized designs are needed to differentiate the relative effects of breathing exercises and respiratory muscle training on physical and psychological outcomes.

The present findings should also be considered alongside other exercise and movement-based interventions that may influence respiratory and functional outcomes in university students. For example, Csepregi et al. reported that classical breathing exercises improved posture, spinal mobility, and chest mobility in female university students and demonstrated benefits comparable to several contemporary training approaches [32]. These findings suggest that respiratory-focused interventions represent one of several potential strategies for improving physical and cardiorespiratory function in young adults. Future studies should directly compare combined breathing exercise and respiratory muscle training programs with aerobic exercise, resistance training, yoga-based interventions, and posture-focused exercise programs to determine their relative effectiveness and feasibility in university settings.

The moderate correlation between TUG and STS (r = 0.49) suggests that improvements in these functional measures may be partially related and could reflect shared adaptations in movement efficiency or neuromuscular function rather than entirely task-specific effects, consistent with prior evidence showing enhanced functional performance following IMT [33].

Sleep quality and perceived stress improved significantly, with mean PSQI scores decreasing from above the threshold indicative of poor sleep quality to below that threshold, indicating meaningful benefits for students. The moderate correlation between improvements in sleep quality and stress (r = 0.33) suggests that these outcomes may be related and could share common underlying mechanisms. One possible explanation is improved autonomic regulation through increased parasympathetic activity and reduced sympathetic tone; however, this mechanism was not directly assessed in the present study [13,16,17]. These findings are consistent with, but do not establish, a model in which autonomic modulation may contribute to improvements in stress, sleep, and functional performance [13,34,35].

The observed improvements in sleep quality and perceived stress are consistent with growing evidence supporting the role of breathing-based interventions in autonomic nervous system regulation. Slow and controlled breathing has been associated with increased vagal activity and reduced sympathetic activation, which may contribute to reductions in perceived stress and improvements in sleep quality [13,15,16]. Respiratory muscle training may further support these adaptations by reducing respiratory effort during daily activities and improving overall breathing efficiency.

Our findings are consistent with previous investigations reporting improvements in respiratory function, exercise tolerance, and psychological outcomes following respiratory training interventions [14,15,28]. The magnitude of change in breath-holding time observed in the present study may reflect the combined use of breathing exercises and threshold-loaded inspiratory and expiratory muscle training.

The moderate correlation between improvements in STS performance and reductions in perceived stress (r = 0.33) suggests a potential relationship between physical and psychological responses to the intervention [13,34]. However, the magnitude of the association was modest, and the underlying mechanisms remain uncertain.

The lack of significant correlations between changes in breath-holding time and other outcomes suggests that improvements in breath-holding performance may represent a distinct aspect of adaptation that was not closely associated with changes in functional performance, sleep quality, or perceived stress in this sample.

PSQI and PSS are self-reported measures and therefore susceptible to expectation effects, reporting bias, and temporal influences unrelated to the intervention. Because both questionnaires assess experiences over approximately the preceding month, responses obtained at post-intervention may partially reflect experiences occurring before and during the intervention period. Data collection occurred during a standard academic semester and not during final examination periods. Nevertheless, variations in academic workload and social stressors could have influenced the observed findings.

The finding that males experienced significantly greater improvements in breath-holding time than females (+28.67 vs. +20.47 s, *p* = 0.023) is intriguing and may reflect baseline anatomical and physiological differences. Males typically have larger lung volumes, greater respiratory muscle mass, and higher baseline MIP values [36], which may provide greater capacity for training-induced adaptation. Alternatively, differences in respiratory muscle fiber type composition or hormonal influences on muscle plasticity could contribute. The absence of gender differences in functional and psychological outcomes suggests that the intervention is equally effective across genders for these domains, though the respiratory-specific difference warrants confirmation in larger studies with objective MIP/MEP measurements.

### 4.1. Study Strengths

This study possesses several notable strengths. First, it addresses an important gap by examining the combined effects of breathing exercises and RMT on both physiological and psychological outcomes in a university student population, with specific relevance to young adults in Saudi Arabia, a context where sedentary lifestyles are increasingly prevalent. Second, the integration of objective functional assessments (breath-holding time, TUG, STS) with validated subjective questionnaires (PSQI, PSS) enabled comprehensive evaluation of physical and psychological outcomes. Third, the use of change scores for correlation analysis allowed us to examine relationships among intervention-induced adaptations while removing confounding by baseline individual differences, providing unique insight into potential shared mechanisms. Fourth, the inclusion of both male and female participants enabled preliminary examination of gender-specific responses. Fifth, the high adherence rate (≥90%) demonstrates the feasibility and acceptability of this intervention in a real-world university setting.

The significant correlations observed between improvements in sleep quality, stress, and functional performance provide preliminary evidence that the intervention may influence multiple interconnected physiological systems simultaneously. However, these correlations should be considered exploratory and hypothesis-generating and do not establish mechanistic relationships.

### 4.2. Limitations

This study has several limitations. We acknowledge that aerobic and resistance exercise remain the primary evidence-based approaches for improving physical fitness and cardiometabolic health in university students. The purpose of the present study was not to position respiratory training as a substitute for conventional exercise but rather as a complementary, low-impact intervention that may be easier to integrate into daily routines for students who face barriers to participation in structured exercise programs. Furthermore, the high adherence observed in this study may have been facilitated by the supervised nature of the intervention. Future studies should examine whether similar benefits can be achieved using home-based or minimally supervised respiratory training protocols and compare their effectiveness directly with traditional exercise interventions.

The use of TUG and STS in a healthy young population may be associated with ceiling effects, potentially limiting their sensitivity to detect meaningful functional changes. Furthermore, all outcome assessments were conducted by the same assessor who was aware of the assessment time point (pre- vs. post-intervention). Consequently, measurement bias and test familiarization effects cannot be excluded. Therefore, improvements in functional outcomes should be interpreted cautiously and confirmed in future randomized controlled studies using blinded outcome assessors and additional objective performance measures.

The absence of a control group prevents definitive causal attribution, and improvements may have been influenced by factors such as learning effects, test familiarity, expectation effects, regression to the mean, changes in academic workload, lifestyle behaviors, sleep routines, or other unmeasured variables. Nevertheless, the short intervention duration, specificity of the observed changes, and consistency across several outcome measures provide preliminary signals that warrant further investigation in controlled studies. Also, the absence of post-intervention MIP and MEP measurements limits direct confirmation of respiratory muscle adaptations. Lack of objective physiological measures (e.g., MIP/MEP, heart rate variability, cortisol) limits mechanistic conclusions. The same non-blinded assessor conducted all tests, introducing potential bias. Self-reported sleep and stress outcomes are subject to recall and expectation bias. The single-university sample of healthy, normal-weight students limits generalizability, and the 4-week follow-up does not address long-term sustainability. Findings should therefore be considered hypothesis-generating and useful for informing the design and sample size estimation of future randomized controlled trials. Further, because participants were healthy and free from respiratory impairment, the potential for physiological improvement may have been limited, and the clinical significance of respiratory muscle training in this population remains to be established.

### 4.3. Practical Implications

The current results are particularly relevant to university students, a population frequently exposed to academic demands, examination-related stress, irregular sleep schedules, and prolonged sedentary behavior. Improvements in sleep quality and perceived stress may therefore have important implications for student well-being, academic engagement, and overall quality of life. Because respiratory interventions require minimal equipment, time commitment, and physical exertion, they may represent a feasible strategy for implementation within university wellness programs.

If confirmed in randomized controlled trials, universities may consider integrating breathing exercises and respiratory muscle training into student wellness and health-promotion programs. Potential implementation strategies include supervised group sessions, incorporation into student orientation programs, home-based practice supported by mobile applications, and inclusion within university health and counseling services.

### 4.4. Future Research

Future randomized controlled trials should include sham control groups, blinded assessors, objective physiological measures such as maximal inspiratory and expiratory pressures, heart rate variability, cortisol, and actigraphy, as well as longer intervention and follow-up periods. Comparative effectiveness studies evaluating respiratory training against conventional exercise interventions are also warranted.

## 5. Conclusions

This pilot single-arm study demonstrated high adherence and feasibility of a four-week combined breathing exercise and respiratory muscle training program among healthy university students. Participation in the program was associated with favorable within-group changes in breath-holding time, functional performance, sleep quality, and perceived stress. Because of the absence of a control group, causal inferences cannot be made. Future randomized controlled trials incorporating sham or control groups, blinded outcome assessment, objective respiratory measures, and longer follow-up periods are needed to determine efficacy and underlying mechanisms.

## Figures and Tables

**Table 1:** Baseline Demographic and Clinical Characteristics of University Students (N = 40).

Characteristic	Value
Age, years	
Median (IQR)	22 (22–23)
Range	19–24
Sex, *n* (%)	
Female	20 (50.0%)
Male	20 (50.0%)
Anthropometric Measurements (Mean ± SD)	
Height, cm	165.85 ± 8.77
Weight, kg	64.47 ± 13.19
BMI, kg/m^2^	23.14 ± 4.05
Muscle Mass, kg	27.51 ± 9.51
Baseline Outcome Measures	
Chest Expansion (cm), Median (IQR)	37.00 (34.90–39.12)
Breath-Holding Time (s), Median (IQR)	21.36 (18.05–27.85)
Timed Up and Go (s), Mean ± SD	8.18 ± 1.12
Sit-to-Stand (s), Mean ± SD	10.12 ± 1.89
Pittsburgh Sleep Quality Index (score), Mean ± SD	5.90 ± 2.99
Perceived Stress Scale (score), Mean ± SD	15.78 ± 6.02

Abbreviations: SD, Standard Deviation; and BMI, Body Mass Index. Values are median (IQR) for age due to non-normal distribution; mean ± SD for other continuous variables.

**Table 2:** Comparison of Pre- and Post-Intervention Outcomes (N = 40).

Variable	Pre-Intervention	Post-Intervention	Difference	Test Statistic	*p*-Value	Effect Size
Chest Expansion (cm) ^a^	37.00 (34.90–39.12)	37.25 (35.00–39.50)	0.00 (−0.50, 1.00)	Z = −1.476	0.140	r = 0.23
Breath-Holding Time (s) ^a^	21.36 (18.05–27.85)	49.48 (42.86–58.58)	+27.12 (21.45, 33.67)	Z = −5.511	<0.001	r = 0.87
Timed Up-and-Go (s) ^b^	8.18 ± 1.12	7.42 ± 1.17	−0.75 (−0.99, −0.51)	t = −6.39	<0.001	d_z = −1.01
Sit-to-Stand (s) ^b^	10.12 ± 1.89	8.90 ± 1.50	−1.21 (−1.56, −0.87)	t = −7.06	<0.001	d_z = −1.12
Pittsburgh Sleep Quality Index ^b^	5.90 ± 2.99	4.35 ± 2.35	−1.55 (−2.43, −0.67)	t = −3.56	0.001	d_z = −0.56
Perceived Stress Scale ^b^	15.78 ± 6.02	12.80 ± 5.34	−2.98 (−4.53, −1.42)	t = −3.88	<0.001	d_z = −0.61
Weight (kg) ^b^	64.47 ± 13.19	64.45 ± 13.01	−0.01 (−0.39, +0.36)	t = −0.08	0.936	d_z = −0.01
BMI (kg/m^2^) ^a^	23.32 (20.32–26.23)	24.05 (20.50–26.42)	+0.10 (−0.30, 0.50)	Z = −1.01	0.312	r = 0.16
Muscle Mass (kg) ^a^	25.55 (20.10–35.42)	24.80 (18.82–32.90)	−0.30 (−1.50, 1.20)	Z = −0.10	0.920	r = 0.02

^a^ Values are median (IQR); analyzed with Wilcoxon signed-rank test; ^b^ Values are mean ± SD; analyzed with paired *t*-test; Difference shows Hodges–Lehmann median (95% CI) for Wilcoxon, mean (95% CI) for *t*-test. r = |Z|/√N (Rosenthal); d = Cohen’s dz.

**Table 3:** Gender Subgroup Analysis of Change Scores.

Outcome Measure	Males (*n* = 20)	Females (*n* = 20)	Test Statistic	*p*-Value	Effect Size
Chest Expansion (cm)	+0.17 ± 0.24	+0.11 ± 1.05	U = 190	0.803	r = 0.05
Breath-Holding Time (s)	+28.67 ± 8.99	+20.47 ± 10.27	U = 284	0.023 *	r = −0.42
TUG (s)	−0.93 ± 0.74	−0.57 ± 0.72	t = −1.60	0.118	d_z = −0.51
Sit-to-Stand (s)	−1.38 ± 0.90	−1.05 ± 1.25	t = −0.94	0.353	d_z = −0.30
PSQI (score)	−1.30 ± 1.78	−1.80 ± 3.50	t = 0.57	0.573	d_z = 0.18
PSS (score)	−3.25 ± 3.09	−2.70 ± 6.22	t = −0.35	0.725	d_z = −0.11

Abbreviations: TUG, Timed Up and Go test; PSQI, Pittsburgh Sleep Quality Index; PSS, Perceived Stress Scale. Values represent mean change scores ± standard deviation (post-intervention − pre-intervention). U = Mann–Whitney U statistic; t = independent-samples t statistic (df = 38); r = rank-biserial correlation coefficient; d = Cohen’s d. Mann–Whitney U tests were used for non-normal distributions and independent-samples *t*-tests for normally distributed variables. * *p* < 0.05.

## Data Availability

The data presented in this study are available on request from the corresponding author due to participant privacy.
